# Thiamine-Mediated Cooperation Between Auxotrophic *Rhodococcus ruber* ZM07 and *Escherichia coli* K12 Drives Efficient Tetrahydrofuran Degradation

**DOI:** 10.3389/fmicb.2020.594052

**Published:** 2020-12-10

**Authors:** Hui Huang, Minbo Qi, Yiming Liu, Haixia Wang, Xuejun Wang, Yiyang Qiu, Zhenmei Lu

**Affiliations:** MOE Laboratory of Biosystem Homeostasis and Protection, Institute of Microbiology, College of Life Sciences, Zhejiang University, Hangzhou, China

**Keywords:** tetrahydrofuran degradation, thiamine auxotroph, cooperation, interaction mechanism, *Rhodococcus ruber* ZM07, *Escherichia coli* K12

## Abstract

Tetrahydrofuran (THF) is a universal solvent widely used in the synthesis of chemicals and pharmaceuticals. As a refractory organic contaminant, it can only be degraded by a small group of microbes. In this study, a thiamine auxotrophic THF-degrading bacterium, *Rhodococcus ruber* ZM07, was isolated from an enrichment culture H-1. It was cocultured with *Escherichia coli* K12 (which cannot degrade THF but can produce thiamine) and/or *Escherichia coli* K12Δ*thiE* (which can neither degrade THF nor produce thiamine) with or without exogenous thiamine. This study aims to understand the interaction mechanisms between ZM07 and K12. We found that K12 accounted for 30% of the total when cocultured and transferred with ZM07 in thiamine-free systems; in addition, in the three-strain (ZM07, K12, and K12Δ*thiE*) cocultured system without thiamine, K12Δ*thiE* disappeared in the 8th transfer, while K12 could still stably exist (the relative abundance remained at approximately 30%). The growth of K12 was significantly inhibited in the thiamine-rich system. Its proportion was almost below 4% after the fourth transfer in both the two-strain (ZM07 and K12) and three-strain (ZM07, K12, and K12Δ*thiE*) systems; K12Δ*thiE*’s percentage was higher than K12’s in the three-strain (ZM07, K12, and K12Δ*thiE*) cocultured system with exogenous thiamine, and both represented only a small proportion (less than 1% by the fourth transfer). The results of the coculture of K12 and K12Δ*thiE* in thiamine-free medium indicated that intraspecific competition between them may be one of the main reasons for the extinction of K12Δ*thiE* in the three-strain (ZM07, K12, and K12Δ*thiE*) system without exogenous thiamine. Furthermore, we found that ZM07 could cooperate with K12 through extracellular metabolites exchanges without physical contact. This study provides novel insight into how microbes cooperate and compete with one another during THF degradation.

## Introduction

As an important solvent, tetrahydrofuran (THF) is widely applied in the chemical industry. According to toxicity tests, THF induces cell proliferation and causes DNA damage, thereby increasing the risk of cancer in laboratory animals (Gamer et al., 2002; Hermida et al., 2006); moreover, severe central nervous system disorders can be triggered by acute exposure to high concentrations of THF (Katahira et al., 1982; Chhabra et al., 1990). In recent years, biodegradation has been universally acknowledged as an ecofriendly remediation strategy for contaminant removal with high degradation rates (Oh et al., 2010). However, a few kinds of THF-degrading microorganisms (including sixteen bacteria and three fungi) have been reported (Bernhardt and Diekmann, 1991; Parales et al., 1994; Kohlweyer et al., 2000; Skinner et al., 2009; Yao et al., 2009; Masuda et al., 2012; Tajima et al., 2012; Sei et al., 2013; Ren et al., 2020). Furthermore, the biodegradation pathway of THF has not been investigated thoroughly (Skinner et al., 2009). One of the most widely accepted THF metabolic pathways is the oxidation pathway, whereby THF is initially oxidized into 2-hydroxytetrahydrofuran (2-OH THF), which then can form γ-butyrolactone or γ-hydroxybutyraldehyde. Both of these compounds can form γ-hydroxybutyrate, which can be further oxidized to succinate and enter the tricarboxylic acid cycle (Bernhardt and Diekmann, 1991; Thiemer et al., 2003; Morenohorn et al., 2005; Tajima et al., 2012).

According to previous studies, bacteria are ubiquitous and coexist to survive in nature (Dubey and Ben-Yehuda, 2011; Schuster et al., 2013; Amin et al., 2015; Bruger and Waters, 2018). Cooperative behaviors among microorganisms are commonplace (Brown and Johnstone, 2001; Velicer, 2003). For microbes, communication and cooperation are essential in their natural lives. Through cooperation, microorganisms can more efficiently perform biological functions, such as foraging, biofilm formation, pathopoiesis, anabolism and biodegradation (Webb et al., 2003; Du et al., 2012; Rakoff-Nahoum et al., 2014; Filkins et al., 2015; Tao et al., 2017). Many crucial genetic functions of cooperators are leaky, some productions are produced by microbes of community and become the “public good,” which represents an important type of cooperation between microorganisms (Morris et al., 2012; Morris, 2015). Not only the producers but also other nearby members can benefit from diffusible substances released into the environment (Van Gestel et al., 2014; Lujan et al., 2015). Cross-feeding (or syntrophy) is another important type of interaction among microorganisms (Sieuwerts et al., 2008; Morris et al., 2013). Metabolites are released into the extracellular environment and then utilized by others as nutrients or energy sources (Mee et al., 2014; Pande et al., 2016). Previous studies found that a number of microorganisms are auxotrophic in nature and rely on external nutrients (i.e., amino acids, vitamins and other cofactors) for growth (D’Souza et al., 2014; Wexler and Goodman, 2017; Liu et al., 2018). Hydrocarbon-degrading bacterial consortia are built by microbial interactions among amino acids and vitamin auxotrophy and their cooperators (Hubalek et al., 2017). However, the question remains as to how auxotrophic microorganisms, especially contaminant-degrading bacteria, perform their functions in microbial communities. In this study, a THF-degrading bacterium, *Rhodococcus ruber* ZM07, was isolated from a THF-degrading bacterial culture H-1 (Huang et al., 2019). This strain cannot be continuously passaged in basal salt medium (BSM) with THF as the sole carbon source, although it can cooperate with many kinds of other bacteria, such as *Escherichia coli* K12, to form a stable symbiotic system. Strain ZM07 has been verified as a thiamine auxotroph. Therefore, it is very likely that some of the microorganisms that degrade refractory pollutants, e.g., THF, but have not yet been isolated might also be auxotrophic and depend on other non-degrading microbes to perform their functions in nature. Strain ZM07 was deemed highly suitable for this study, which investigated this interesting phenomenon and the ecological significance of auxotrophic strains.

Cooperative behavior can potentially be exploited by uncooperative cheaters (Fiegna and Velicer, 2003; West et al., 2006), which gain benefits from other cooperators without contributing; thus, they achieve a competitive advantage and can invade the community (Smith, 2001; Velicer, 2003; West et al., 2006). If cheaters are unrestricted in the bacterial community, they may quickly overgrow, ultimately leading to a population collapse of the community (Hardin, 1968). In a previous study, cheaters could help to counteract competition among microorganisms and foster biodiversity in well-mixed media by invading the community of cooperating siderophore producers (Leinweber et al., 2017). However, another study indicated that cheaters could be resisted by the conditional privatization strategy under stressful conditions (Jin et al., 2018). Therefore, it might be important to investigate the behavior pattern of uncooperative organisms in systems. We could deduce the stability of the cooperative community of auxotrophic degrading bacteria and nondegrading bacteria in actual environments, such as in wastewater treatment, by studying the noncooperator K12Δ*thiE* constructed in this study.

Auxotrophic microorganisms that degrade refractory pollutants depend on other nondegrading microbes to perform their functions in nature. However, the understanding of the interaction mechanisms between degrading and nondegrading microorganisms as well as the ecological significance of auxotrophic strains is limited. In this study, different cooperative and competitive systems were constructed to explore the interspecies and transspecies communications among the thiamine auxotrophic THF-degrading bacterium ZM07 and its cooperator K12 as well as the stability of the cooperative community of these two strains. This research was proposed to investigate the functions of auxotrophic degrading bacteria and nondegrading bacteria in a cooperative system for THF degradation. The proposed model of auxotrophic THF-degrading and non-THF-degrading microorganisms may provide an insight into the interaction mechanisms in multispecies ecosystems.

## Materials and Methods

### Strains and Plasmids

The strains and plasmids used in this study are listed in Table 1. The THF-degrading strain *Rhodococcus ruber* ZM07 (collection number CCTCC AB 2019217) was isolated from an enrichment culture designated H-1 (Huang et al., 2019). The non-THF-degrading strain *Escherichia coli* K12 (Bachmann et al., 1976) and its knockout strain K12Δ*thiE* (which can neither degrade THF nor produce thiamine) were both cocultured with ZM07, and the latter strain was constructed as described in the Supplementary Methods. The λ-Red recombination plasmid pDK46 (Datsenko and Wanner, 2000) used for *thiE* disruption was purchased from Youbio Biological Technology Co., Ltd. (Changsha, China). Plasmid pGemT7cat (Król et al., 2010) was used as a template for PCR amplification of the fused chloramphenicol-selectable marker.

**TABLE 1:** Strains and plasmids used in this study.

Strain or plasmid	Relevant characteristics	References or source
**Strains**		
*Rhodococcus ruber* ZM07	THF-degrading, non-thiamine-synthesizing strain G^+^	This study
*Escherichia coli* K12	Wild-type, non-THF-degrading, thiamine-synthesizing strain, G^–^	Bachmann et al., 1976
*Escherichia coli* K12Δ*thiE*	*Escherichia coli* K12 mutant with *thiE* gene replaced by chloramphenicol resistance gene from plasmid pGemT7cat, Chl^R^	This study
**Plasmids**		
pDK46	λ Red recombinase expression, Amp^R^	Datsenko and Wanner, 2000
pGemT7cat	Source of chloramphenicol resistance gene	Król et al., 2010

### Subculture Conditions and Experiments

The strains were initially cultivated in lysogeny broth (LB) culture medium and then transferred into BSM supplemented with THF as the sole carbon source. One liter of LB contained 10 g tryptone, 5 g yeast extract, and 10 g NaCl, and the initial pH of the media was 7.4. One liter of BSM contained 3.240 g K_2_HPO_4_, 1.000 g NaH_2_PO_4_⋅H_2_O, 2.000 g NH_4_Cl, 0.123 g C_6_H_8_NNa_3_O_7_, 0.200 g MgSO_4_⋅7H_2_O, 0.012 g FeSO_4_⋅7H_2_O, 0.003 g MnSO_4_⋅H_2_O, 0.003 g ZnSO_4_⋅7H_2_O, and 0.001 g CoCl_2_⋅6H_2_O (Parales et al., 1994; Huang et al., 2019). For experiments under thiamine-rich conditions, 0.01 mM external thiamine was added to the BSM. All strains used in this study were cultured in 100 mL of BSM with an initial pH of 7.0 at 30°C, referred to the conditions of THF-degrading bacterial culture H-1 (Huang et al., 2019). Most experiments were performed using 500 mL shaking flasks at 160 rpm, while specifically designed two-phase reactors were used in the experiment to verify the interaction mode between strains ZM07 and K12 with a shaking rate of 100 rpm.

(1) To investigate the interaction mechanism between ZM07 and K12, this study used a two-phase reactor that can separate cells but allow extracellular metabolites to pass through. The reactor is divided into two chambers (each 500 mL by volume and containing 100 mL of BSM) by track-etched polyethylene terephthalate (PET) nucleopore membranes (with a pore size distribution at 0.22 μm), which were purchased from Wuwei Kejin Xinfa Technology Co., Ltd. (Gansu, China). The test experiments of the reactor performance are detailed in Supplementary Methods. Strains ZM07 and K12 were inoculated separately into each side of the reactor at a ratio of 1:1 (the initial inoculum size of each strain was OD_600_ = 0.06). Samples were collected every 24 h for analysis of the biomass (OD_600_) and determination of the residual THF concentrations.
(2) Coculture of ZM07 and K12: Strains were cultivated in LB, harvested by centrifugation (7,000 × g, 10 min), and washed with BSM three times. The resulting cells were resuspended with BSM (OD_600_ = 3); subsequently, 100 mL of BSM with 20 mM THF was inoculated with 1 mL of strain ZM07 and 1 mL of strain K12 to generate a cell ratio of 1:1 for coculture as the first transfer. The corresponding monoculture systems were incubated with the same inoculum of ZM07 and 1 mL of BSM. Subsequently, the coculture and monoculture were transferred (OD_600_ = 6 and 1 mL of cell suspension) in the same way for the second and third transfers. Samples were collected every 24 h for analysis of the biomass (OD_600_) and determination of the residual THF concentrations.
(3) Coculture of K12 and K12Δ*thiE*: To test the survivability of K12Δ*thiE* in the thiamine-limited and thiamine-rich media used in this study, we cocultured K12 wild type and K12Δ*thiE* in BSM supplemented with 20 mM succinate as the sole carbon source. K12 and K12Δ*thiE* were inoculated with a cell ratio of 1:1 either supplemented with (0.01 mM) thiamine or without thiamine. We used the method described above to passage coculture strains for 8 transfers every 12 h, and the initial cell densities and cell ratios of K12 and K12Δ*thiE* are shown in Supplementary Table S1. Samples were collected at the end of cultivation of each transfer for analysis of biomass (OD_600_) and relative abundance.
(4) Coculture of ZM07, K12, and K12Δ*thiE*: To assess the synergetic and competitive relationships in the three-strain (ZM07, K12, and K12Δ*thiE*) and two-strain (ZM07 and K12, ZM07 and K12Δ*thiE*) communities during THF degradation with or without extra thiamine, we conducted experiments on three different combinations of the three strains (ZM07 and K12, ZM07 and K12Δ*thiE*, ZM07 and K12 and K12Δ*thiE*) with sufficient (0.01 mM) thiamine or without thiamine. The initial inoculum size and strain ratio of the three strains in every experiment are shown in Supplementary Table S1. Each combination with thiamine was transferred to fresh medium every 3 days, and 2 mL samples were collected at 48 h for analysis of the biomass (OD_600_) and determination of the residual THF concentrations and relative strain abundances. Analogously, the cultures without additional thiamine were transferred every 4 days, and samples were collected at 72 h for analysis of the biomass (OD_600_) and determination of the residual THF concentrations and relative strain abundances.

### Determining the Composition of Different Strains in Coculture Systems

The relative abundances of ZM07, K12, and K12Δ*thiE* were determined by quantitative PCR (qPCR). Genomic DNA of the bacterial consortium was isolated with an E.Z.N.A^®^ Bacterial DNA Kit (Omega Bio-Tek) according to the manufacturer’s instructions. Primers for the qPCR assay are shown in Supplementary Table S2. The qPCR reactions were carried out in a volume of 20 μL containing 10 μL of TB Green^TM^ Premix Ex Taq^TM^ (TaKaRa, Dalian, China), 0.4 μL each of forward and reverse gene-specific primers (10 μM), 0.4 μL of DNA (50–100 ng/μL) and 8.8 μL of ddH_2_O. The following conditions were used for PCR: 95°C for 2 min, followed by 40 cycles of 10 s at 95°C, 20 s at 58°C and 20 s at 72°C. Three independent DNA samples were assayed, and the 2^–ΔΔCT^ method was used to calculate the gene abundance level of the strains (Livak and Schmittgen, 2001).

### KEGG Metabolic Pathways Analysis

The metabolic pathways of strains ZM07 and K12 were analyzed using the method described in previous research (Li et al., 2018). The whole-genome sequencing results of strains ZM07 and K12 were annotated by the Kyoto Encyclopedia of Genes and Genomes (KEGG^1^). The draft genome sequence data of strain ZM07 have been included in the National Center for Biotechnology Information (NCBI), accession number JACVXT000000000, and strain K12 genome sequence data can also be found in NCBI (GenBank accession number NC_000913.3). We then compared the results to find differences and annotated them in KEGG Mapper with different colors (pathways that are unique in ZM07 and K12 are colored green and blue, respectively, while pathways harbored by both strains are colored brown). We searched for the anabolic pathways that were completely present only in the K12 strain.

### Verification of Thiamine Auxotroph

Experiments were conducted to verify that strain ZM07 is a thiamine auxotroph. Different amounts of thiamine were added to the third transfer of ZM07 (the strain could barely grow in BSM with 40 mM THF as the sole carbon source after continuous passage culture). Furthermore, strain ZM07 was cocultured with K12 and K12Δ*thiE* with 40 mM THF, respectively, for two transfers. Samples were collected 72 h after each transfer for analysis of the biomass (OD_600_) and determination of the residual THF concentrations.

### Invasion Experiments With Trace Amounts of K12 in Microbial Communities

To verify whether trace wild-type K12 can form a stable interaction system with ZM07, we added 0.5% (OD_600_ = 0.0003) K12 wild type to ZM07 culture in the first transfer and passaged the culture for 8 transfers. In addition, we added 0.5% of the K12 wild type to the third transfer of the ZM07 culture, which was cultivated for 4 days alone with almost no growth. In other tests, 0.5% K12Δ*thiE* was added to the ZM07 and K12 coculture in the first and third transfers and passaged for 8 transfers to determine how trace K12Δ*thiE* behaves when invading the ZM07 and K12 system. The initial cell densities and cell ratios of the three strains in every experiment are shown in Supplementary Table S1. Each combination was transferred to fresh medium every 4 days, and samples were collected at 72 h for analysis of the biomass (OD_600_) and determination of the residual THF concentrations and relative strain abundances.

### Intermediate Metabolites of THF Used to Culture Strain K12

Strain ZM07 in the first transfer was cultured in 100 mL of BSM supplemented with 20 mM THF for 1, 2, 3, and 4 days. Then, the supernatant of ZM07, which was obtained using a 0.22 μm vacuum bottle filter (BIOFIL, China), was used to cultivate strain K12 (the initial inoculum size of K12 was OD_600_ = 0.06) for 3 days. Furthermore, THF, 2-OH THF, γ-butyrolactone and succinate (three intermediate metabolites of THF) were chosen to test the ability of strain K12 to use these compounds (5 mM each). Strain K12 was cultured in 100 mL of BSM at 30°C and 160 rpm, with samples collected after 12, 24, and 72 h to measure the OD_600_. Furthermore, the effects of THF, 2-OH THF and γ-butyrolactone on the growth and morphology of K12 and K12Δ*thiE* were also studied as described in Supplementary Methods.

### Detection and Identification of THF and Related Metabolites and Statistical Analysis

The cultures were centrifuged at 10,000 × g for 10 min, and 500 μL of the supernatant was subjected to THF concentration determination using gas chromatograph (GC-2014C; SHIMADZU, Japan). The temperatures of the injector, oven, and detector were set to 250, 160, and 250°C, respectively. The degradation ratio was calculated as described in our previous study (Huang et al., 2019). The THF intermediate metabolites produced by strain ZM07 were detected as described in Supplementary Methods. The biomass was monitored by recording the OD_600_ using a UV spectrometer (UV-3100PC; MAPADA; Shanghai). Succinate detection is shown below: Samples of extracellular supernatants and intracellular extracts were derivatized using pyridine and N-methyl-N-(trimethylsilyl) trifluoroacetamide (MSTFA) and analyzed by gas chromatography-mass spectrometry (GC-MS). Each experiment was performed in triplicate. *P*-values for all assays were determined using a two-tailed Student’s *t*-test.

## Results

### KEGG Metabolic Pathway Analysis Revealed That the THF-Degrading Bacterium ZM07 Is a Thiamine Auxotroph Strain

**FIGURE 1:**
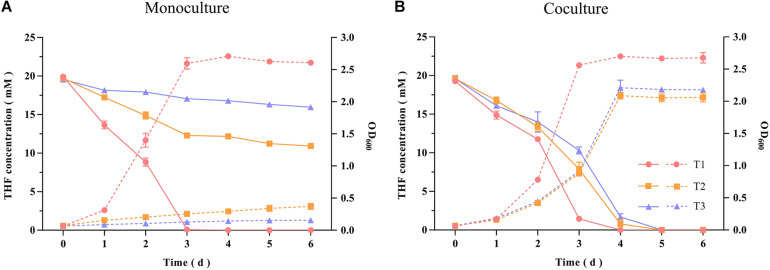
THF degradation and growth curves of strain ZM07 monocultured **(A)** and cocultured with strain K12 **(B)** in the first, second and third transfers. The solid lines represent THF concentration curves; the dotted lines represent growth curves (OD_600_); the circles represent the first transfer; the squares represent the second transfer; and the triangles represent the third transfer. Error bars: SD from three independent replicates, which may be smaller than the marker.

The THF-degrading strain *Rhodococcus ruber* ZM07 was isolated from an enrichment culture H-1 (Huang et al., 2019), and cannot be subcultured serially in BSM with THF as the sole carbon source. Before carrying out experiments, monoculture growths of strains ZM07, K12, and K12Δ*thiE* in BSM (not supplemented with THF or any other carbon sources) with or without thiamine were tested. The results showed that these three strains cannot grow in BSM without THF or any other carbon sources (Supplementary Figure S1). Monoculture experiments showed that ZM07 degraded 20 mM THF completely within 3 days in the first transfer, while its THF-degrading ability was inhibited when its growth rate slowed in the second transfer; furthermore, its growth was inhibited when using THF in the third transfer (Figure 1A). We speculated that intracellular thiamine, which may still remain during the first transfer, supported the growth of strain ZM07 in the starting phase. In contrast, when cocultured with strain K12, ZM07 was able to proliferate well and continually degrade THF during passaging (Figure 1B).

**FIGURE 2:**
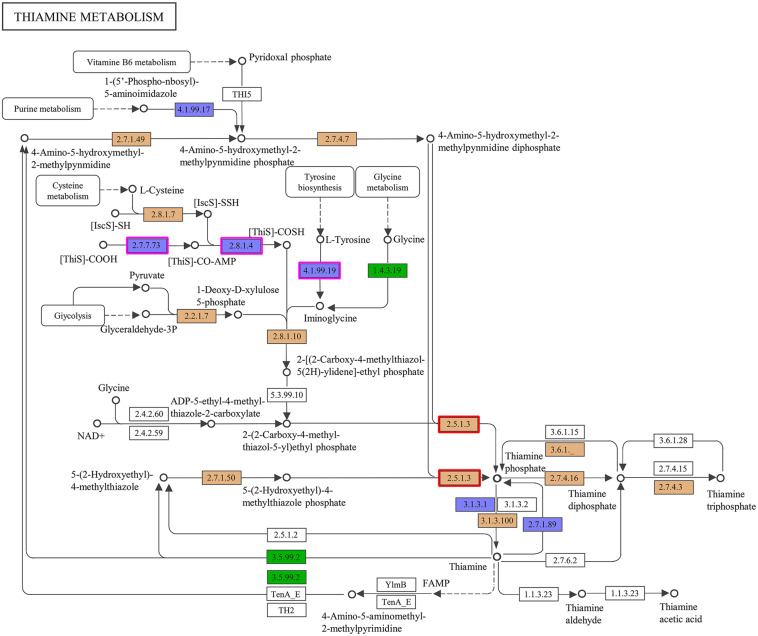
Comparison of the KEGG metabolic pathways reconstructed from the complete genomes of ZM07 and K12. Here, thiamine metabolism (map00730) is shown. The ZM07-specific pathways are colored in green; the K12-specific pathways are colored in blue; shared pathways are colored in brown; and pathways present in neither ZM07 nor K12 are colored in white. The genes for *thiF* (No. 2.7.7.73), *thiH* (No. 4.1.99.19), and *thiI* (No. 2.8.1.4) are highlighted by purple boxes, and the gene for *thiE* (No. 2.5.1.3) is highlighted by red boxes.

Accordingly, we suspected that the THF-degrading bacterium ZM07 may not be able to produce some growth factors that are critical to its growth. To verify this hypothesis, we plotted a comparative metabolic pathway network for ZM07 and K12 with the pathways harbored by the different strains, which are colored differently (Supplementary Figure S2). The map of thiamine metabolism supports our hypothesis since the thiamine synthesis pathway is incomplete in strain ZM07 but intact in strain K12 (Figure 2). As an essential growth factor, thiamine pyrophosphate is produced through the condensation reaction of 4-amino-5-hydroxymethyl pyrimidine pyrophosphate and 4-methyl-5-(beta-hydroxyethyl)-thiazole monophosphate (Begley et al., 1999). As shown in Figure 2, strain ZM07 lacks the genes *thiF*, *thiH*, and *thiI* (highlighted by the purple boxes), which are required for 4-methyl-5-(beta-hydroxyethyl)-thiazole synthesis (Webb et al., 1997; Dorrestein et al., 2004; Martinez-Gomez et al., 2004).

To further verify our hypothesis, we performed two experiments. First, we cocultured the wild-type strain K12 and the *thiE* (highlighted by the red boxes in Figure 2)-deficient strain K12Δ*thiE* separately with strain ZM07. The *thiE* gene encodes thiamine phosphate synthase (ThiE), which is responsible for linking the thiazole and pyrimidine moieties of thiamine monophosphate (TMP) generated in respective metabolic pathways (Chiu et al., 1999; Jurgenson et al., 2009). The results showed that strain ZM07 could grow and degrade THF normally when cocultured and transferred with K12 wild type (Supplementary Figure S3D), while it could not restore its growth and THF degradation ability when cocultured with K12Δ*thiE* (which is very obvious after the second transfer) (Supplementary Figure S3E). The coculture of ZM07 and K12Δ*thiE* (basically no growth) quickly became cloudy with additional thiamine on the 1st day, with a final OD_600_ of 2.541 ± 0.056 (data not shown), while the growth of coculture without additional thiamine was very slow (Supplementary Figure S4A). Further experiments were performed to confirm that THF-degrading bacterium ZM07 is indeed a thiamine auxotroph strain by adding thiamine directly to the culture medium. The results showed that when supplied with more than 10^–5^ mM thiamine, strain ZM07 grew well and degraded THF normally (Supplementary Figure S5). Nevertheless, the growth and THF-degrading ability of ZM07 were still inhibited when thiamine concentrations were below 10^–5^ mM (Supplementary Figure S5). The results indicated that THF-degrading bacterium ZM07 cannot grow alone with THF as the sole carbon source since it is a thiamine auxotroph strain.

### Contact-Independent Interaction Mode Between Strains ZM07 and K12

To determine the interaction mode between strains ZM07 and K12, the metabolic intermediates of THF were detected and identified at different cultivation time of strain ZM07 when utilizing 20 mM THF as substrates firstly. The results showed that no metabolites were detected in extracellular supernatants (Supplementary Figure S6A), while one peak that appeared at retention times of 6.816 min were observed in intracellular extracts (Supplementary Figure S6B). According to the retention times of standards, the sources of the peak was identified as γ-butyrolactone. Furthermore, succinate was identified in both of the samples of extracellular supernatants and intracellular extracts (Supplementary Figures S6D,E). To investigate whether K12 could utilize the intermediate metabolites of THF degraded by ZM07 in the cocultured system, we used THF and its intermediate metabolites (2-OH THF, γ-butyrolactone and succinate) as carbon sources to cultivate strain K12. The results showed that K12 could utilize only succinate to survive, with the highest OD_600_ value reaching 0.305 ± 0.002, while it could not utilize THF, 2-OH THF, or γ-butyrolactone as sole carbon sources (Supplementary Table S3). The effects of several different concentrations (0, 1.25, 2.5, and 5 mM) of THF, 2-OH THF and γ-butyrolactone on growth of K12 and its mutant were tested. The results showed that different THF, 2-OH THF, and γ-butyrolactone concentrations (0, 1.25, 2.5, and 5 mM) did not significantly influence the biomass of K12 or K12Δ*thiE* (Supplementary Figure S7). Otherwise, the transmission electron microscopy (TEM) images of two strains morphology after exposuring the cell to these three compounds indicated that there are no obvious changes of both two strains exposing to 5 mM THF, 2-OH THF, and γ-butyrolactone (Supplementary Figure S8).

**FIGURE 3:**
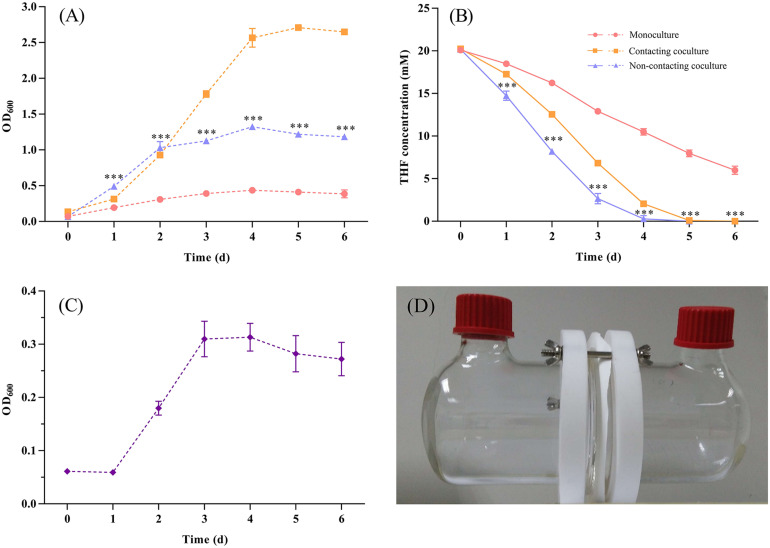
**(A)** Growth curves of strain ZM07 monocultured (pink line) and cocultured with K12 under the non-contact condition (blue line) in two-phase reactors and the growth curve of the two strains cocultured under the contact condition (yellow line). **(B)** THF degradation curves of strain ZM07 in the three cases described above. **(C)** Growth curve of strain K12 cocultured with ZM07 under the non-contact condition in a two-phase reactor. **(D)** Two-phase reactor used to separate the cultured strains in this study. The *P*-value indicates a significant difference between monocultured strain ZM07 and strain ZM07 cocultured with K12 in two-phase reactors and was determined using Student’s *t*-test (*n* = 3, ^∗∗∗^*p* < 0.001). Error bars: SD from three independent replicates, which may be smaller than the marker.

In addition, we used culture supernatants of ZM07 (cultured for 1, 2, 3, and 4 days) to culture strain K12 (Supplementary Table S4), and it showed no growth probably because THF intermediate metabolites in the supernatants of strain ZM07, which could be used by strain K12, are easily accessible carbon sources and cannot be accumulated, hence the instantaneous concentration is too low to support the growth of K12. Finally, two-phase reactors were used to explore whether physical contact was needed to mediate the interaction between these two species. The test experiments of the reactor performance indicated that the membrane can separate cells and allow only extracellular metabolite exchange (Supplementary Figures S9, S10). The results showed that the separately cultured strain K12 could help ZM07 grow and degrade THF (Figures 3A,B). A comparison with the contacting coculture of ZM07 and K12 showed that THF was degraded even faster in the group of the non-contacting coculture (Figure 3B), which indicated that there might be some cooperation and competition mechanisms in these two strains. The highest OD_600_ value of K12 reached 0.313 ± 0.026 (Figure 3C), indicating that physical contact is not a prerequisite for the interaction between strains ZM07 and K12. Separately cultured strain K12 could grow well when continuous supplying with THF intermediate metabolites as carbon sources from ZM07 through membrane, while it was not growing with discontinuous supplies cultured in the supernatants of strain ZM07. Thus, the exchange of extracellular metabolites is one way these two strains interact.

### Intraspecific Competition Between K12 and K12ΔthiE

**FIGURE 4:**
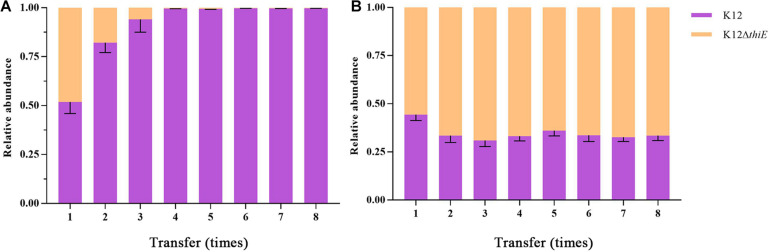
Relative abundances of K12 and K12Δ*thiE* cocultured under 20 mM succinate without **(A)** or with **(B)** 0.01 mM thiamine across different transfers. Error bars: SD from three independent replicates.

Succinate, one of the intermediate metabolites of THF, was used as a carbon source to explore the intraspecific competition between wild-type K12 and its knockout strain. The results showed that under thiamine-limited conditions, strain K12 wild type outcompeted strain K12Δ*thiE*. The proportion of K12Δ*thiE* was drastically reduced during passage and disappeared almost entirely by the fourth transfer (Figure 4A), while its proportion remained stable in the coculture system under thiamine-rich conditions. Moreover, the composition of K12Δ*thiE* and K12 remained stable (at a ratio of approximately 6:4) during passage with sufficient thiamine (Figure 4B). However, there was no difference in the biomass between K12 and K12Δ*thiE* with or without thiamine in the first transfer (Supplementary Figures S11A,B). Furthermore, strain K12Δ*thiE* cannot be continuously passaged in BSM without thiamine, and the final biomass of 1 day of cultivation between K12 and the coculture of K12 and K12Δ*thiE* showed no difference (Supplementary Figure S11C). In conclusion, K12Δ*thiE* has obvious disadvantages in thiamine-limited medium. However, additional thiamine can significantly increase the competitiveness of K12Δ*thiE* against its wild-type relative.

### Competition and Cooperation Among ZM07, K12, and K12ΔthiE in Thiamine-Limited Medium

**FIGURE 5:**
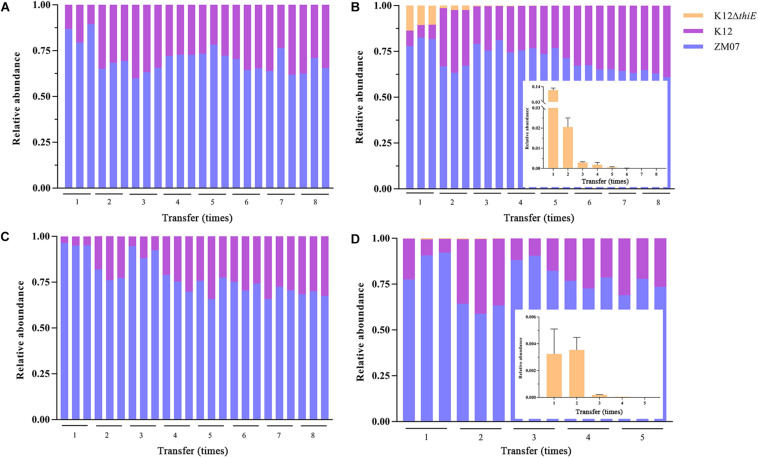
Relative abundances of ZM07, K12, and K12Δ*thiE* in a two-strain system (ZM07 and K12) **(A)** and in a three-strain system **(B)** across different transfers without thiamine. Relative abundances of three strains in a two-strain system (ZM07 and trace amount of K12) **(C)** and in a three-strain system (ZM07, K12 and trace amount of K12Δ*thiE*) **(D)** across different transfers. The plots within **(B,D)** show details of the relative abundance of K12Δ*thiE* in corresponding experiments. Three independent replicates were performed for each group. Error bars: SD from three independent replicates.

In thiamine-limited medium, experiments revealed that the relative abundance of K12 remained at approximately 30% in both the two-strain system (ZM07 and K12) and the three-strain system (ZM07, K12, and K12Δ*thiE*) during passage without additional thiamine (Figures 5A,B). In the invasion experiments, a trace amount (the initial inoculum size of the strain was OD_600_ = 0.0003, and same as below) of K12 was added to the ZM07 culture in the 1st transfer, and the results showed that the relative abundances of ZM07 and K12 finally became similar to those of the coculture of ZM07 and K12 without additional thiamine (Figure 5A) during passage (Figure 5C). In addition, the addition of trace amounts of K12 to the third transfer of the ZM07 culture that was cultivated for 4 days alone with almost no growth could easily restore the growth and THF-degrading ability of ZM07 (Supplementary Figure S4B). These results indicated that strain K12 could help auxotrophic strain ZM07 rapidly regain its growth and THF degradation abilities. Then, the results for the coculture of ZM07, K12, and K12Δ*thiE* showed that K12Δ*thiE* could not continuously exist in the symbiotic system composed of ZM07 and K12 in thiamine-limited medium and instead disappeared after the 8^th^ transfer (Figure 5B). In the invasion experiments, when trace amounts of K12Δ*thiE* were added to the ZM07 and K12 system in the 1^st^ transfer, K12Δ*thiE* disappeared by the 8^th^ transfer during passage (Figure 5D). Additionally, to eliminate the influence of intracellular thiamine, which may still remain during the first two transfers, experiments were performed in which trace amounts of K12Δ*thiE* were added to the ZM07 and K12 system in the 3^rd^ transfer; however, K12Δ*thiE* disappeared even faster (Supplementary Figure S12). These results indicated that K12Δ*thiE* cannot invade the symbiotic system of strains ZM07 and K12.

### Competition and Cooperation Among ZM07, K12, and K12ΔthiE in Thiamine-Rich Medium

**FIGURE 6:**
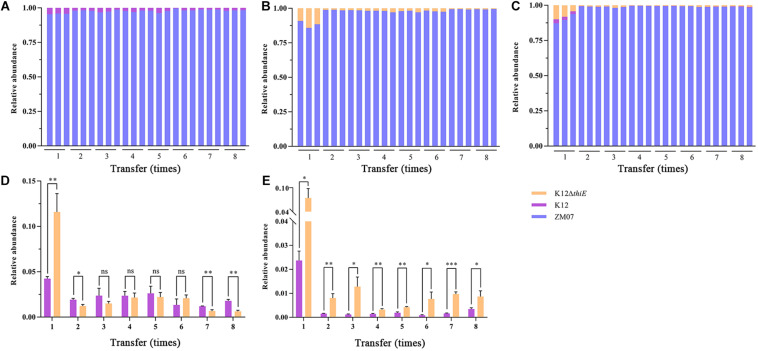
Relative abundances of ZM07, K12, and K12Δ*thiE* in two-strain systems (ZM07 and K12) **(A)**, (ZM07 and K12Δ*thiE*) **(B)** and a three-strain system **(C)** in different transfers with 0.01 mM thiamine. **(D)** Relative abundances of K12 and K12Δ*thiE* in the above experiments in two-strain systems (the relative abundance of strain K12 in a cocultured system of ZM07 and K12 in **(A)** and the relative abundance of strain K12Δ*thiE* in a cocultured system of ZM07 and K12Δ*thiE* in **panel (B)** in different transfers with 0.01 mM thiamine. **(E)** Relative abundances of K12 and K12Δ*thiE* in the above experiments in the three-strain system (ZM07, K12, and K12Δ*thiE* in **(C)** in different transfers with 0.01 mM thiamine. Three independent replicates were performed for each group. Error bars: SD from three independent replicates. The *P* value indicates statistical significance between K12 and K12Δ*thiE* determined using Student’s *t*-test (*n* = 3, ^∗^*p* < 0.05, ^∗∗^*p* < 0.01, ^∗∗∗^*p* < 0.001).

In thiamine-rich medium, the results showed that additional thiamine drastically increased the advantage of ZM07 and decreased the proportion of K12 in the coculture of ZM07 and K12 (Figure 6A), which indicated that strain K12 was significantly inhibited by additional thiamine. Moreover, despite occupying only a small proportion of the cocultured systems, strain K12Δ*thiE* existed throughout the whole transfer process in the two-strain system of ZM07 and K12Δ*thiE* (Figure 6B) or in the three-strain system of ZM07, K12, and K12Δ*thiE* (Figure 6C) with additional thiamine, which differed from its fate in thiamine-limited systems (Figure 5B). Furthermore, the proportion of K12 in the cocultured system of ZM07 and K12 showed no significant differences from the proportion of K12Δ*thiE* in the cocultured system of ZM07 and K12Δ*thiE* in thiamine-rich medium (Figure 6D); however, K12Δ*thiE* grew better than K12 in the three-strain system with additional thiamine (Figure 6E), which was similar to the results of the intraspecific competition experiment of K12 and K12Δ*thiE* (Figure 4B). The results indicated that additional thiamine could increase the competitiveness of K12Δ*thiE* against its wild-type relative. Additionally, the THF degradation ratio and the total biomass of the systems showed no significant difference among different groups (monoculture of ZM07; two-strain coculture of ZM07 and K12, or ZM07 and K12Δ*thiE*; and three-strain coculture of ZM07, K12, and K12Δ*thiE*) in thiamine-rich medium (Supplementary Figure S13), and all combinations of these three strains mentioned above were able to completely degrade 20 mM THF within 4 days. We can conclude that exogenous thiamine might eliminate the effect of K12 and K12Δ*thiE* on the THF degradation ability of strain ZM07 in the cocultured systems.

## Discussion

Auxotrophic strains rely on external nutrients for growth, and they are also very likely to benefit their cooperators, which facilitates the establishment of a stable interaction system between them. Rhodococci have a wide range of degradation spectra (Martínková et al., 2009), compatibility with foreign genes (Larkin et al., 2006), and effective degradability; therefore, they have enormous application potential for the treatment of environmental pollutants. Thiamine auxotrophic microorganisms are ubiquitous in the *Rhodococcus* genus (Hummel et al., 1987; Denome et al., 1994). The *Rhodococcus ruber* strain ZM07 used in this study is a natural thiamine auxotrophic THF-degrading strain. Compared with other strains in the enrichment culture H-1, strain ZM07 seems to have greater survival advantages in nutrient-poor environments with high THF concentrations (Huang et al., 2019). Therefore, during the long-term evolution process, why have many species of *Rhodococcus* genus lost thiamine synthesis ability, even though it is extremely important for their survival and they cannot live independently without a supply of thiamine provided by other microbes? The “Black Queen Hypothesis” and streamlining theory might provide a reference for this topic; according to these theories, genome reduction might result in higher fitness and optimized resource allocation (Morris et al., 2012; Wolf and Koonin, 2013; Batut et al., 2014; Giovannoni et al., 2014; Otero-Bravo et al., 2018; Zengler and Zaramela, 2018). *Prochlorococcus* mutants gradually lost the ability to reduce HOOH in water; however, they can still enjoy the benefits of decomposing H_2_O_2_ because other microorganisms in the community still have this function (Morris et al., 2012). Accordingly, we conjecture that “landlords,” such as the strain ZM07 and *Prochlorococcus* mutants, may play vital roles in the microbial community. Their lost functions can be compensated for by “leakage” from synthesis-capable organisms since thiamine, a public good, is a readily available substance in the environment. In this manner, the auxotrophic functional bacterium ZM07 may be able to degrade THF more effectively and easily by forming a stable symbiotic system with non-THF-degrading bacteria in nature.

According to previous studies, cooperation through public good is ubiquitous among microbial members in nature (Crespi, 2001; West et al., 2006). In this study, we proposed a contact-independent interaction mode between ZM07 and K12. Based on the results of two-phase reactors, these two strains’ cooperation relies on extracellular metabolic interactions, which do not require physical contact between strains (Figure 3). During THF degradation, cooperator K12 provides thiamine as a public good to ZM07, which is critical to growth and could pass through the membrane without difficulty (Supplementary Figure S10). Through the results of THF intermediate metabolites detection and using abilities of K12, THF-degrading bacterium ZM07 most likely provided available metabolites of THF that are easily used, such as succinate for K12 (Supplementary Table S3 and Supplementary Figure S6). In addition, the highest OD_600_ value of ZM07 cultured separately with K12 in two-phase reactors reached only 1.323 ± 0.022 (Figure 3A), while it could reach up to 2.708 ± 0.012 with normal growth in the first transfer (Figure 1A). The low public goods exchange rate between the two strains might represent an important reason for its poor growth. Alternatively, there might be some other contact-dependent interaction mechanisms between these two strains that need to be further studied. However, cooperators (who produce public goods) would generate a metabolic cost, while all the microorganisms in the community benefit from it, regardless of whether they are producers (Brockhurst et al., 2010; Popat et al., 2012). Hence, such cooperative systems can easily be invaded by cheaters. The invasion of cheaters usually causes low proportions of cooperators who contribute public goods to maintain growth stability, consequently damaging the interests of the cooperative system (Dunny et al., 2008). In the cocultured system of ZM07, K12, and K12Δ*thiE* in thiamine-limited medium, the non-cooperator K12Δ*thiE* did not cause the entire community to crash; instead, it gradually disappeared during the passages (Figure 5B), indicating that the symbiotic system of strains ZM07 and K12 was stable and K12Δ*thiE* could not invade it. The reasons for the success of the cooperative system that resists the non-cooperator might be as follows. First, intraspecific competition experiments between the K12 wild type and defective strain showed that under thiamine-limited conditions, K12Δ*thiE* faced fierce intraspecific competition and was inhibited during coculture with K12 (Figure 4). Intraspecific competition experiments indicate that one of the reasons for the failure of K12Δ*thiE* is the insufficient thiamine secreted by K12 to the culture medium. A previous study showed that thiamine tends to be stored intracellularly rather than secreted extracellularly (Schyns et al., 2005), which also supports our conjectures. Furthermore, the results showed that the growth of strain ZM07 was inhibited by the shortage of thiamine in the community (Supplementary Figure S5) and that the degrading bacterial strain ZM07 cannot keep the entire community functioning with such low activity in the thiamine-limited medium. At the beginning of inoculation, strain ZM07 lacked thiamine, strain K12 lacked a carbon source, and both strains depended on each other to grow; hence, it was impossible to eliminate either of the two strains through rapid proliferation, which eventually might result in a stable symbiotic system. Experiments using trace amounts of K12 to rapidly restore the growth and THF-degrading ability of ZM07 (Figure 5C) also demonstrated the stability of the community of ZM07 and K12. In the cocultured system, strain ZM07 might have stronger thiamine utilization ability than K12Δ*thiE*, and since it can degrade THF, strain ZM07 could gain some preferential access to the broken-down products. These two reasons might explain why strain ZM07 shows a faster growth rate than K12Δ*thiE*, which might be an important factor in resisting non-cooperator invasion. In the fierce competition for dual nutrients (carbon source and thiamine), the non-cooperator could not find a sufficient niche in the coculture system without additional thiamine. Moreover, the dual pressures of carbon deficiency and thiamine deficiency might have accelerated K12Δ*thiE* extinction.

In this study, the addition of thiamine weakened the advantage of K12, whether it was cocultured with ZM07 alone or cocultured with both ZM07 and K12Δ*thiE*, and the relative abundance of K12 was decreased to a very low level in the cocultured systems (Figures 6A,C). Otherwise, no difference occurred in the proportions of K12 and K12Δ*thiE* when they were cocultured separately with ZM07 in thiamine-rich medium (Figure 6D), indicating that adding thiamine eliminates the advantage of the K12 wild type, which can only attain its position when needed by ZM07 in a cocultured system. Why could the cooperative system recognize the role of K12 and inhibit it under thiamine-rich conditions? We speculate that the THF-degrading bacterium ZM07 might reduce the secretion of intermediate metabolites of THF when thiamine is easily available. Conversely, strain ZM07 has to maintain its THF-degrading ability through a metabolite cross-feeding interaction with K12 under thiamine-limited conditions. Furthermore, K12Δ*thiE* grew better than K12 in the three-strain system with additional thiamine (Figure 6E), indicating that there is a metabolic cost for thiamine synthesis as discussed above. In the three-strain communities in this study, the non-cooperator K12Δ*thiE* disappeared by the 8th transfer in thiamine-limited medium (Figure 5B) but remained in the medium supplemented with thiamine (Figure 6C), indicating that the additional thiamine might slow down the elimination of K12Δ*thiE* in cooperative communities. Accordingly, in the kingdom of the THF degradation system, cooperators may utilize thiamine as “rent” that they exchange for “food” (carbon sources) from the “landlord,” the THF-degrading bacterium. Non-cooperators who are unable to provide “rent” are likely to be gradually eliminated.

**FIGURE 7:**
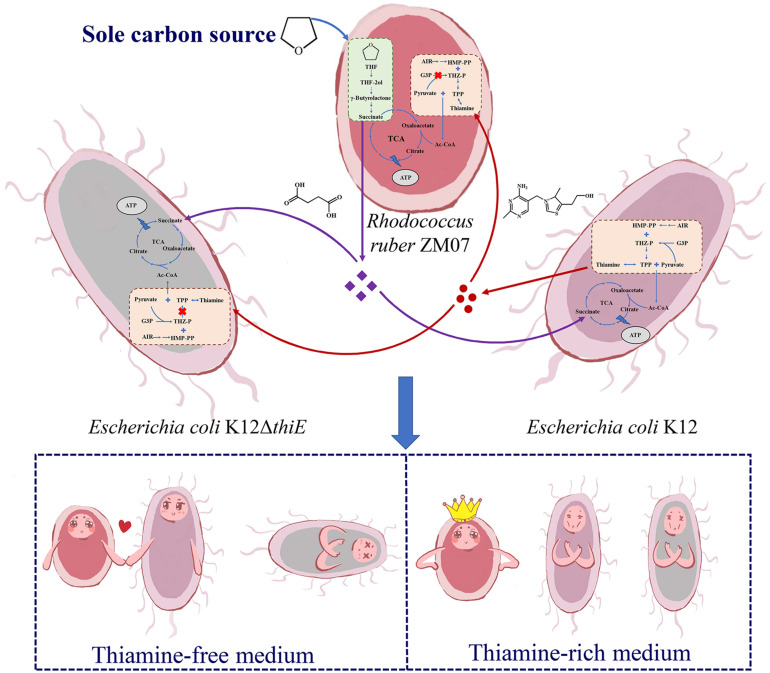
Proposed schematic depiction of the interaction mechanism among strains ZM07, K12, and K12Δ*thiE* during THF degradation. Strain K12 provides thiamine (red circles) to ZM07 and K12Δ*thiE*, and strain ZM07 might provide succinate or other downstream products (purple diamonds) to K12 and K12Δ*thiE* as a carbon source by degrading THF. In thiamine-free medium, ZM07 and K12 formed a stable symbiotic system, and K12Δ*thiE* disappeared during passaging; in thiamine-rich media, both K12 and K12Δ*thiE* represented only a small proportion of the coculture system.

Based on the above results, we propose a model depicting the succession process of these three strains during passaging in the communities. In a cocultured system, K12 provides thiamine, an essential growth factor, as a public good to thiamine auxotrophic bacteria ZM07 and K12Δ*thiE*; THF-degrading bacterium ZM07 degrades THF (the sole carbon source in the cocultured system) and provides easily accessible intermediates (succinate and other downstream products) to the cooperator strain K12 and the non-cooperator K12Δ*thiE* as carbon sources. Eventually, the non-cooperator K12Δ*thiE* (which can neither degrade THF nor produce thiamine) disappears during passaging, indicating that non-cooperator strain K12Δ*thiE* cannot invade the symbiotic ZM07 and K12 system (Figure 7). In thiamine-rich medium, both K12 and K12Δ*thiE* represented only a small proportion of the coculture system, while ZM07 became dominant (Figure 7). Based on the observed interactions between strains ZM07 and K12, “rent” must be paid to maintain a place in the symbiotic system of THF degradation; non-cooperators who cannot pay the “rent” might be excluded by the cooperative system. However, thiamine auxotrophic THF-degrading bacterium ZM07 might not need cooperators when surrounded by sufficient thiamine. Taken together, we show that cooperation and competition mechanisms exist in THF multispecies ecosystems to maintain the stability of communities. Auxotrophic degrading bacteria and their cooperators can play an important role in species coexistence in microbial communities.

## Data Availability Statement

The raw data supporting the conclusions of this article will be made available by the authors, without undue reservation.

## Author Contributions

ZL and HH conceived and designed the experiments. HH and YL performed the experiments. HH, MQ, and YL analyzed the data. HH, HW, XW, and YQ wrote the manuscript. All authors agreed to be accountable for all aspects of the work in ensuring that questions related to the accuracy or integrity of any part of the work are appropriately investigated and resolved.

## Conflict of Interest

The authors declare that the research was conducted in the absence of any commercial or financial relationships that could be construed as a potential conflict of interest.

## Footnotes

1 www.genome.jp/kegg
