# Supplementary material for: Thiamine-Mediated Cooperation Between Auxotrophic Rhodococcus ruber ZM07 and Escherichia coli K12 Drives Efficient Tetrahydrofuran Degradation
Source: Front Microbiol. 2020 Dec 10;11:594052. doi: 10.3389/fmicb.2020.594052 (PMC7758286; doi:10.3389/fmicb.2020.594052)
Supplement: Supplementary file 1 [file Data_Sheet_1.docx]

Supplementary Material

**SUPPLEMENTARY METHODS**

## Construction of *Escherichia coli* K12Δ*thiE*

A *thiE* gene (encoding a synthase in the thiamine synthesis pathway) knockout *E. coli* K12 strain was constructed using λ-Red recombination methods. To replace the *thiE* gene along with a chloramphenicol-selectable marker in the chromosome, we used PCR-mediated λ-Red recombination with a PCR fragment containing the fused chloramphenicol resistance gene and two upstream and downstream fragments of the target genes. Linear DNA fragments of the chloramphenicol resistance gene were generated by PCR from pGemT7cat with 5′- and 3′-primer pairs (**Table** **S1)**. The underlined sequences represent homology arms (50 bp), while the bold entries are the priming sequences for hybridization to the complementary nucleotide sequences on the template DNA (*cat*). Standard PCR conditions were used to amplify the linear DNA fragment with *TransStart**^®^ FastPfu* DNA Polymerase (TransGen Biotech, Beijing, China). The resulting PCR products were further purified and extracted using the E.Z.N.A.^®^ Gel Extraction Kit (Omega Bio-Tek).

Plasmid pDK46 was transformed into *E. coli* K12 before the preparation of electrocompetent cells. The methods of electrocompetent cell preparation and electroporation were similar to those used in [35]. Additionally, we added 1 mL of cold LB medium after electroporation, and the electrocompetent cells were further incubated at 37°C for 2 h, coated on LB culture medium plates containing 25 μg/mL chloramphenicol, and incubated at 37°C overnight to select chloramphenicol-resistant recombinants. K12Δ*thiE* was verified by PCR and sequencing analysis (data not shown); test primers are shown below (**Table S1**). Sequencing was performed by Tsingke Biological Technology Co., Ltd. (Beijing, China).

## The test experiments of the two-phase reactor performance

The test experiments are shown below: Firstly, strain K12 was inoculated into one side of the reactor with 100 mL LB, and the other side without bacteria. The culture of the side without bacteria of the reactor was observed after three days of cultivation. Secondly, we tested whether thiamine and succinate can pass through the membrane. We filled the reactor with 100 mL of BSM, and added thiamine and succinate to one chamber of it, samples of the other chamber were collected before and after one day of shaking (with the same shaking condition we described in the manuscript). High performance liquid chromatography (HPLC) was used to test the thiamine standard and samples. Succinate standard and samples were derivatized using pyridine and N-Methyl-N-(trimethylsilyl) trifluoroacetamide (MSTFA) and identified by gas chromatography-mass spectrometry (GC-MS) analysis.

## Effects of THF, 2-OH THF and γ-butyrolactone on growth of strains K12 and K12Δ*thiE*

Impacts of different THF, 2-OH THF, and γ-butyrolactone concentrations on growth curves of strains K12 and K12Δ*thiE* were examined. Two strains were respectively cultured in LB medium with different concentrations (0, 1.25, 2.5, and 5 mM) of these three compounds for 2 days. Samples were collected every 3 h for analysis of the biomass (OD_600_).

Strains K12 and K12Δ*thiE* were respectively incubated in LB medium with 5 mM THF, 2-OH THF, and γ-butyrolactone, and they were also cultured without any compounds as the control for 24 h. The fresh bacteria were collected and uniformly suspended in a water drop. After that, a copper grid was dipped in the cell suspension and dried with filter paper. The samples were stained using 3% uranyl acetate solution and dried with an infrared lamp. The samples were observed with a JEOL JEM-1230 transmission electron microscope (JEOL, Tokyo, Japan) with guidance.

## Detection and identification of THF intermediate metabolites produced by strain ZM07

Strain ZM07 was cultured in BSM with 20 mM THF as the sole carbon source for 24 h, 36 h, 48 h, 60 h, and 72 h. The supernatants and cell extracts were collected for the detection of metabolites by gas chromatography (GC). The detection program was as follows: the initial temperature was set at 60°C, gradually increased to 160°C at a rate of 20°C min^−1^, and then held at 160°C for 5 min.

**SUPPLEMENTARY TABLES**

**Table S1** Initial inoculum size (OD_600_) and initial ratios of ZM07, K12 and K12Δ*thiE* in the different experiments in this study.

|  | ZM07 | K12 | K12Δ*thiE* | Initial ratio | Experiments |
| --- | --- | --- | --- | --- | --- |
| 1 | 0 | 0.03 | 0.03 | 0:1:1 | Coculture of K12 and K12Δ*thiE* without thiamine (**Figure 4A**) |
| 2 | 0 | 0.03 | 0.03 | 0:1:1 | Coculture of K12 and K12Δ*thiE* with thiamine (**Figure 4B**) |
| 3 | 0.03 | 0.03 | 0 | 1:1:0 | Coculture of ZM07 and K12 without thiamine (**Figure 5A**) |
| 4 | 0.03 | 0.15 | 0.15 | 2:1:1 | Coculture of ZM07, K12 and K12Δ*thiE* without thiamine (**Figure 5B**) |
| 5 | 0.03 | 0.0003 | 0 | 100:1:0 | Coculture of ZM07 and trace amount of K12 without thiamine (**Figure 5C**) |
| 6 | 0.03 | 0.03 | 0.0003 | 100:100:1 | Coculture of ZM07, K12 and trace amount of K12Δ*thiE* without thiamine (K12Δ*thiE* were added from the first transfer) (**Figure 5D**) |
| 7 | 0.03 | 0.03 | 0.0003 | 100:100:1 | Coculture of ZM07, K12 and trace amount of K12Δ*thiE* without thiamine (K12Δ*thiE* were added from the third transfer) (**Figure S11**) |
| 8 | 0.03 | 0.03 | 0 | 1:1:0 | Coculture of ZM07 and K12 with thiamine (**Figure 6A**) |
| 9 | 0.03 | 0 | 0.03 | 1:0:1 | Coculture of ZM07 and K12Δ*thiE* with thiamine (**Figure 6B**) |
| 10 | 0.03 | 0.15 | 0.15 | 2:1:1 | Coculture of ZM07, K12 and K12Δ*thiE* with thiamine (**Figure 6C**) |

**Table S2** Primers used for PCR amplification in this study.

| Primers | Sequence 5′ – 3′ | Purpose |
| --- | --- | --- |
| *ThiE*-*cat*-F | AACTTCCGTGCCAGAGGCGGAGAAATCTACCTGCGTAAGGAGGAAGCGTG**GTGTCCCTGTTGATACCG** | Amplified the universal transfer constructs and obtained linear target segments |
| *ThiE*-*cat*-R | GAGCGATATCGTCGAGCAGGATTTGGCGGCTATAACGCATAAAGTCACGG**GCGTTTAAGGGCACCAAT** |  |
| *ThiE*-full-F/R | CCAGACCGATAATCAGCA/ATGGGCGACTTTACCTGA | Verified successful integration of the PCR product into K12 DNA by PCR analysis |
| *ThiE*-up-F/R | CCAGACCGATAATCAGCA/CGGTATCAACAGGGACAC |  |
| *ThiE*-down-F/R | GCGTTTAAGGGCACCAAT/ATGGGCGACTTTACCTGA |  |
| *Thm*-F/R | CGAGTTCATCCAAGTAGT/GAAGTCTCTATCCACTGAT | Quantified ZM07, K12 and K12Δ*thiE* |
| *ThiE*-F/R | CGACACATGACGATATGG/GAAGGCATCTGTTTGGTT |  |
| *Cat*-F/R | CGTAAGAGGTTCCAACTT/ATTGAGCAACTGACTGAA |  |

The underlined sequences represent homology arms (50 bp), while the bold entries are the priming sequences for hybridization to the complementary nucleotide sequences on the template DNA (*cat*).

**Table S3** Growth profiles of K12 in the presence of THF, 2-hydroxytetrahydrofuran, γ-butyrolactone and succinate (5 mM each) as carbon sources after 12 h, 24 h and 72 h of cultivation.

| Substrate | 12 h | 24 h | 72 h |
| --- | --- | --- | --- |
| Negative control | 0.074±0.002 | 0.073±0.001 | 0.067±0.002 |
| THF | 0.071±0.001 | 0.068±0.001 | 0.064±0.002 |
| 2-Hydroxytetrahydrofuran | 0.062±0.001 | 0.062±0.001 | 0.058±0.001 |
| γ-Butyrolactone | 0.074±0.003 | 0.068±0.000 | 0.064±0.000 |
| Succinate | 0.305±0.002*** | 0.274±0.001*** | 0.181±0.095*** |

The *P* value indicates statistically significant differences between the control and experimental groups determined using Student’s *t* test (*n* = 3, ****p* < 0.001), SD from three replicates.

**Table S4** Growth profiles of K12 cultured in the supernatants of strain ZM07 cultured after 1, 2, 3 and 4 days, with the bacteria removed using a 0.22-μm vacuum bottle filter. And succinate concentrations of the samples of different supernatants.

| Growth time of ZM07 (d) | Final OD_600_ of ZM07 | Initial OD_600_ of K12 | Final OD_600_ of K12 | Succinate (mM) |
| --- | --- | --- | --- | --- |
| Negative control | - | 0.070±0.001 | 0.055±0.003 | 0 |
| 1 | 0.386±0.015 | 0.071±0.001 | 0.040±0.003 | 0.059±0.003 |
| 2 | 1.615±0.026 | 0.072±0.002 | 0.057±0.000 | 0.019±0.002 |
| 3 | 2.839±0.085 | 0.074±0.003 | 0.066±0.002 | 0.034±0.001 |
| 4 | 2.673±0.043 | 0.073±0.002 | 0.067±0.002 | 0.048±0.002 |

SD from three replicates.

**SUPPLEMENTARY FIGURES**

**Figure S1** (A) Growth curve of strain ZM07 in BSM without exogenous thiamine; (B) Growth curve of strain ZM07 in BSM with 0.01 mM thiamine; (C) Growth curve of strain K12 (purple line) and K12Δ*thiE* (yellow line) in BSM without exogenous thiamine; (D) Growth curve of strain K12 and K12Δ*thiE* in BSM with 0.01 mM thiamine. Error bars: SD from three independent replicates.


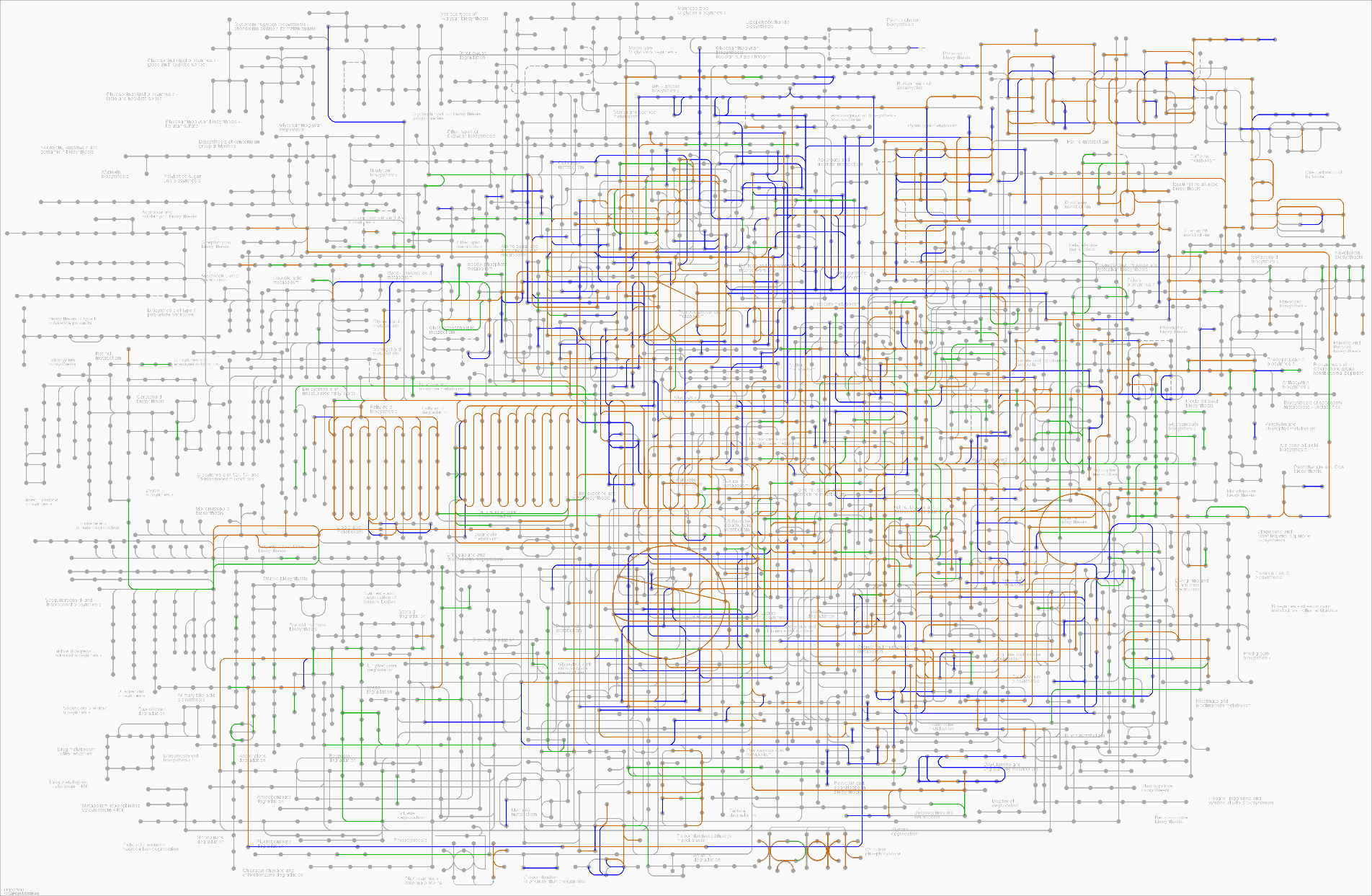


**Figure S2** Map of strain ZM07- and strain K12-related genes in the total metabolism pathway by KEGG mapper. Green represents the genes unique to strain ZM07, blue represents the genes unique to strain K12, and brown represents the genes in common.


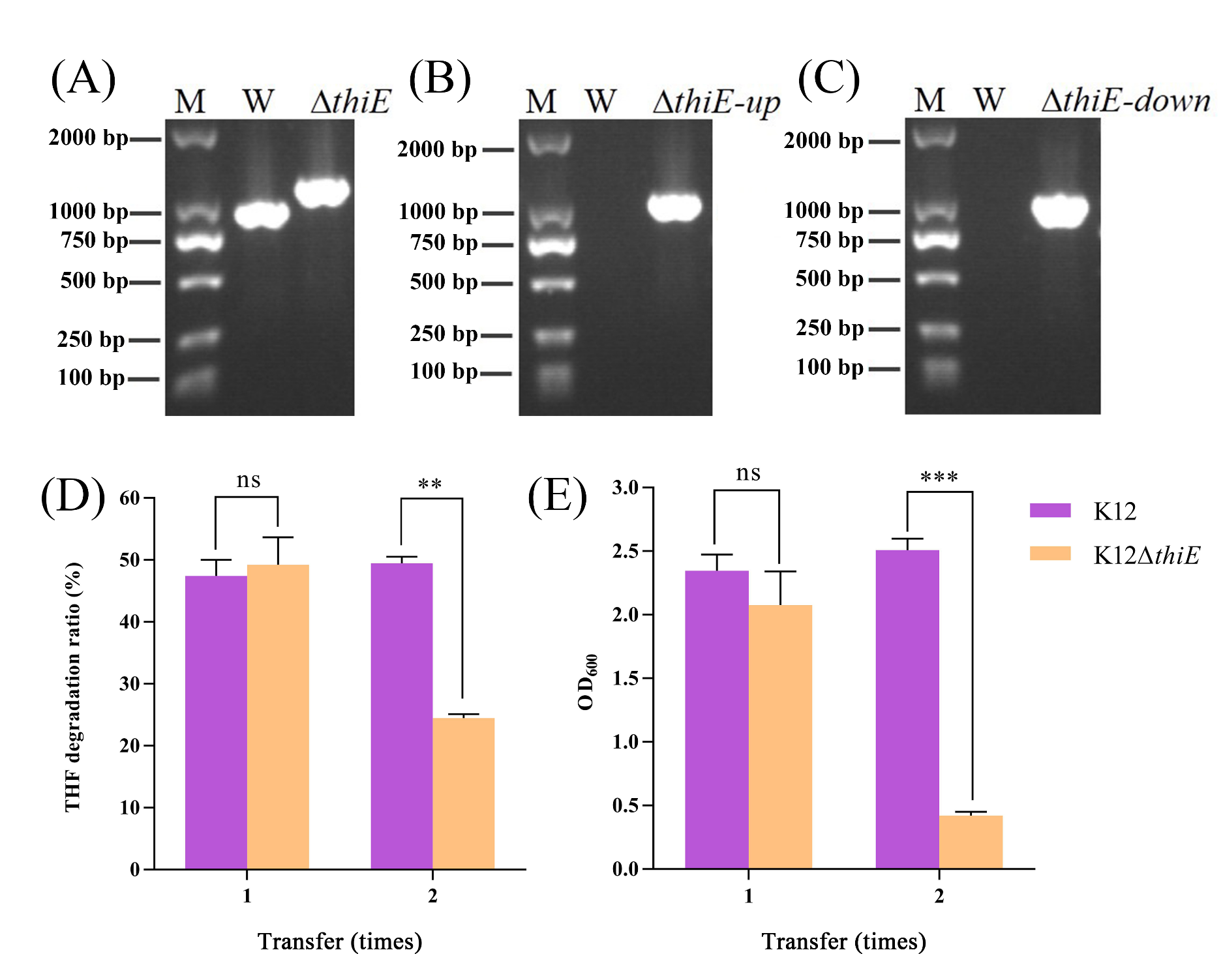


**Figure S3** PCR verification of K12Δ*thiE* using specific primers in Table 1. (A) Full length of *thiE* in wild type (W) and *cat* in K12Δ*thiE* (Δ*thiE*); (B) upstream regions of the *thiE* and a part of *cat*; (C) a part of *cat* and the downstream regions of *thiE*; M, DL2000 marker. Electrophoresis was performed using 0.8% agarose. THF degradation ratio (D) and OD_600_ (E) of ZM07 cocultured with K12 and K12Δ*thiE* for 3 days with 40 mM THF in the first and second transfers. Significance was analyzed by Student’s *t*-test (*n* = 3, ns: *p* > 0.05, **: *p* < 0.01, ***: *p* < 0.001).


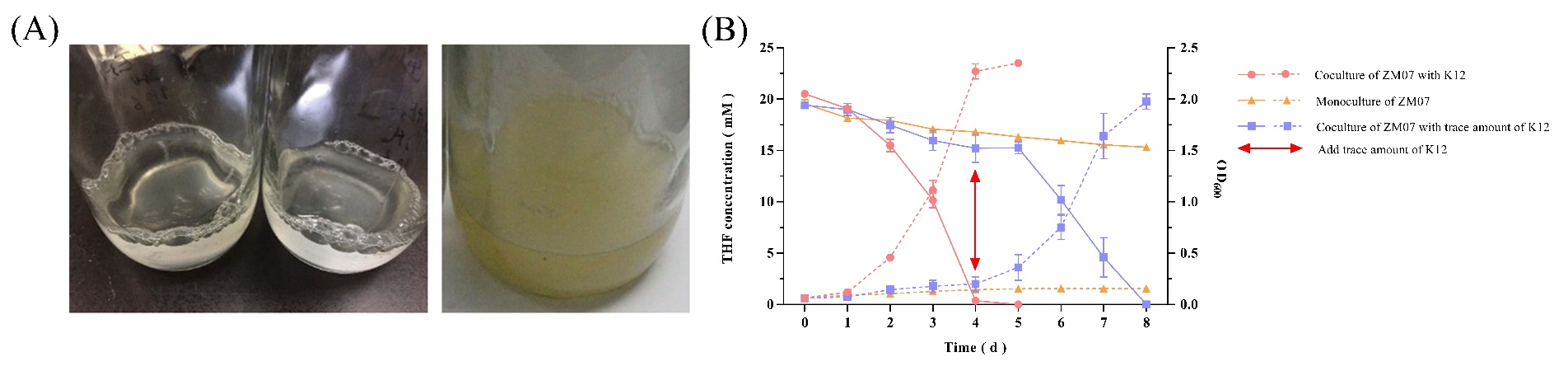


**Figure S4** (A) Coculture of ZM07 and K12Δ*thiE* in the second transfer (left picture) and the coculture after the addition of supplemental thiamine for 1 day (right picture). (B) THF degradation curves and growth curves for ZM07 with K12. The solid lines represent THF concentration curves, and the dotted lines represent growth curves (OD_600_); trace amounts of K12 were added in the third transfer of the ZM07 culture that was cultivated alone for 4 days with almost no growth. Error bars: SD from three independent replicates; error bars may be smaller than the marker.


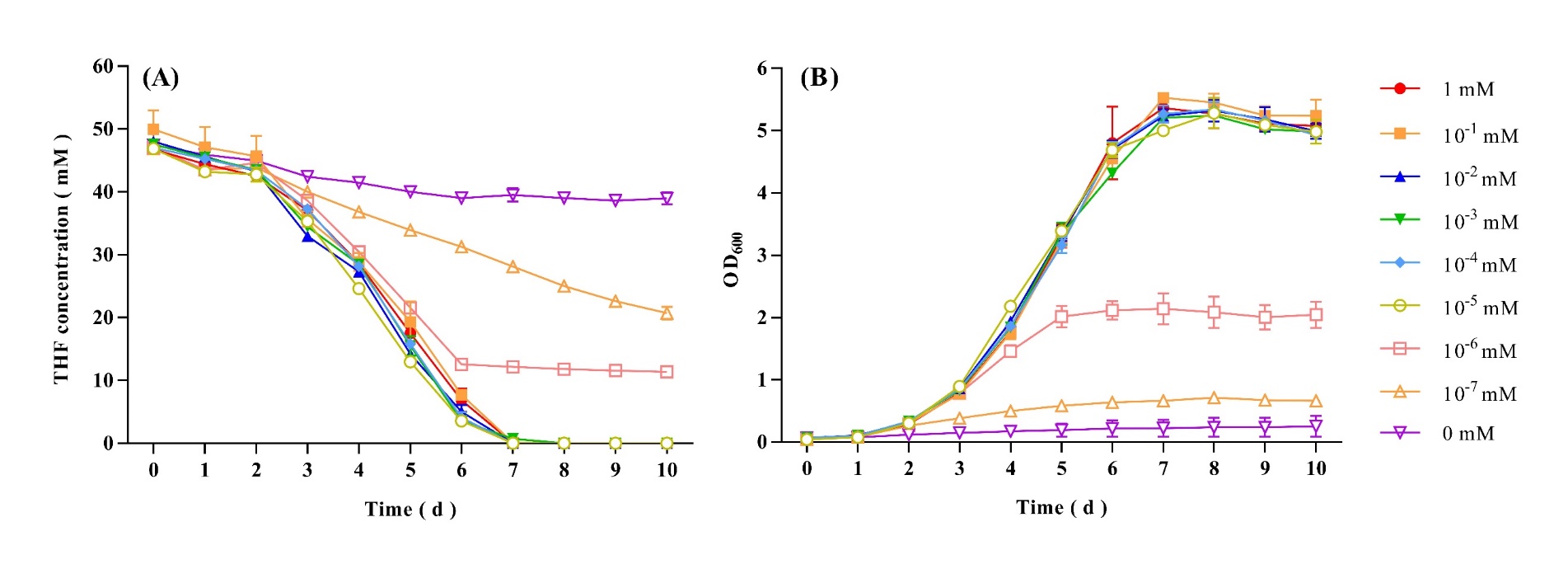


**Figure S5** THF degradation curves (A) and growth curves (B) of strain ZM07 at thiamine concentrations ranging from 0 to 1 mM. Error bars: SD from three independent replicates.


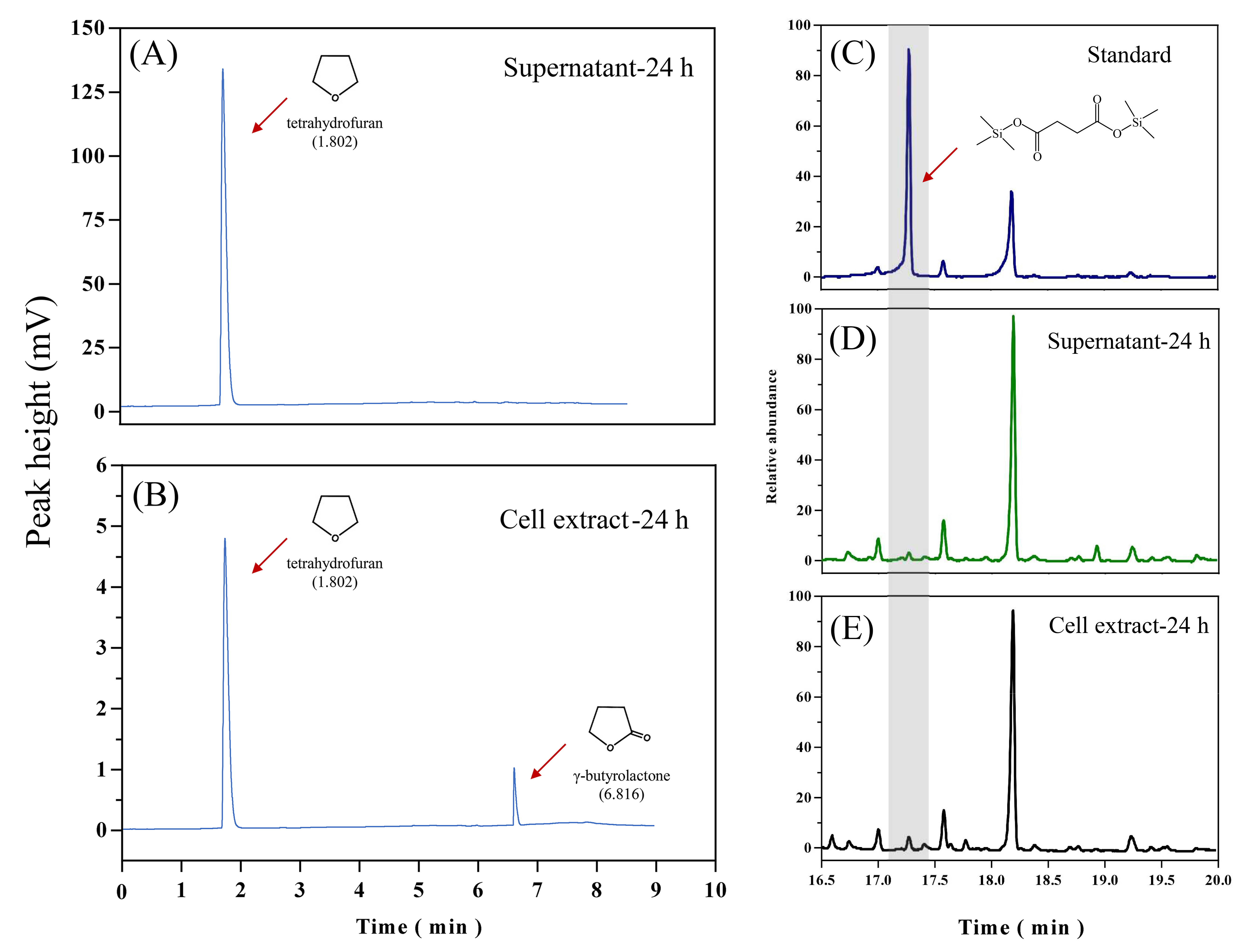


**Figure S6** GC analysis of the supernatants (A) and cell extracts (B) of 24 h sample, and there were the same peaks in 36 h, 48 h, 60 h, and 72 h samples. GC analysis of the succinate standard (C), and the supernatants (D) as well as cell extracts (E) of 24 h sample, and there were the same peaks in 36 h, 48 h, 60 h, and 72 h samples.

**Figure S7** Effects of different concentrations (0, 1.25, 2.5, and 5 mM) of THF (A and B), 2-OH THF (C and D), and γ-butyrolactone (E and F) on biomass (OD_600_) of strains K12 (A, C and E) and K12Δ*thiE* (B, D and F) in LB medium. Error bars: SD from three independent replicates; error bars may be smaller than the marker.


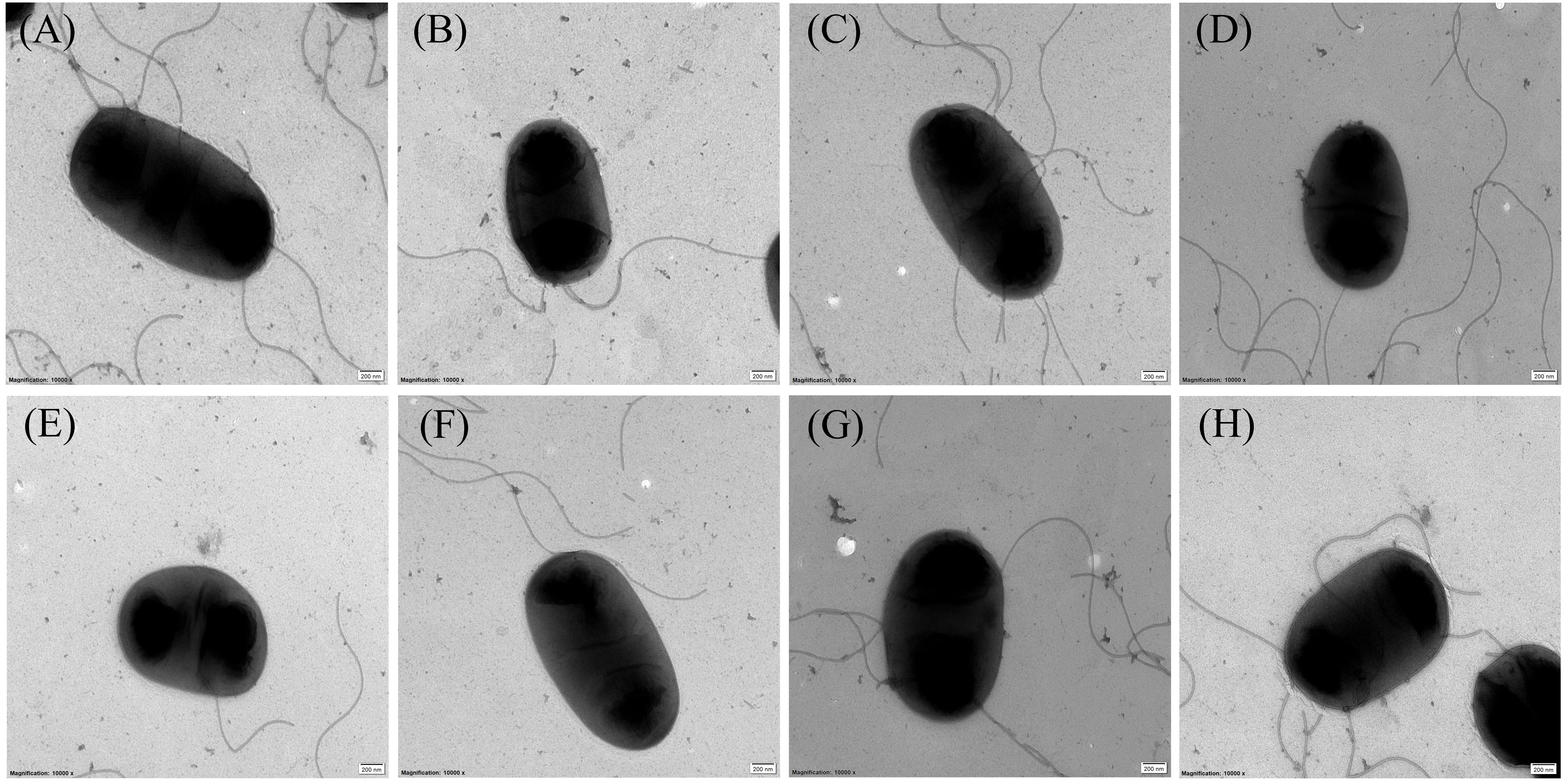


**Figure S8** Transmission electron microscopy (TEM) images of strains K12 (A, B, C, and D) and K12Δ*thiE* (E, F, G and H) exposed to 5 mM THF (B and F), 2-OH THF (C and G), and γ-butyrolactone (D and H) in BSM. Figures A and E represent control groups of K12 (A) and K12Δ*thiE* (E).


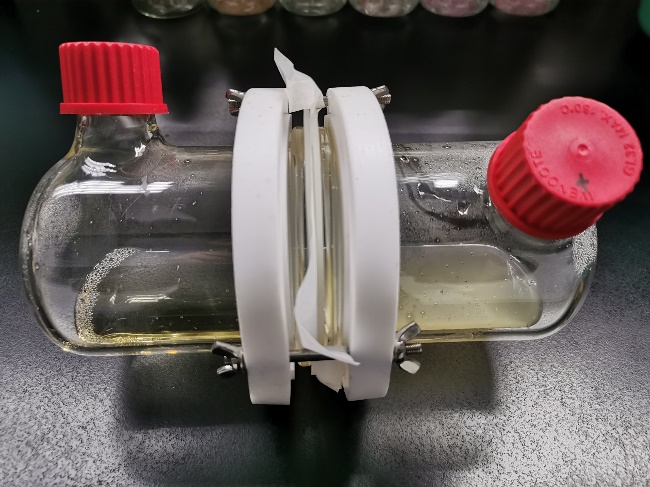


**Figure S9** Two-phase reactor test experiment. Strain K12 was only added into the right side and incubated with shaking for three days.


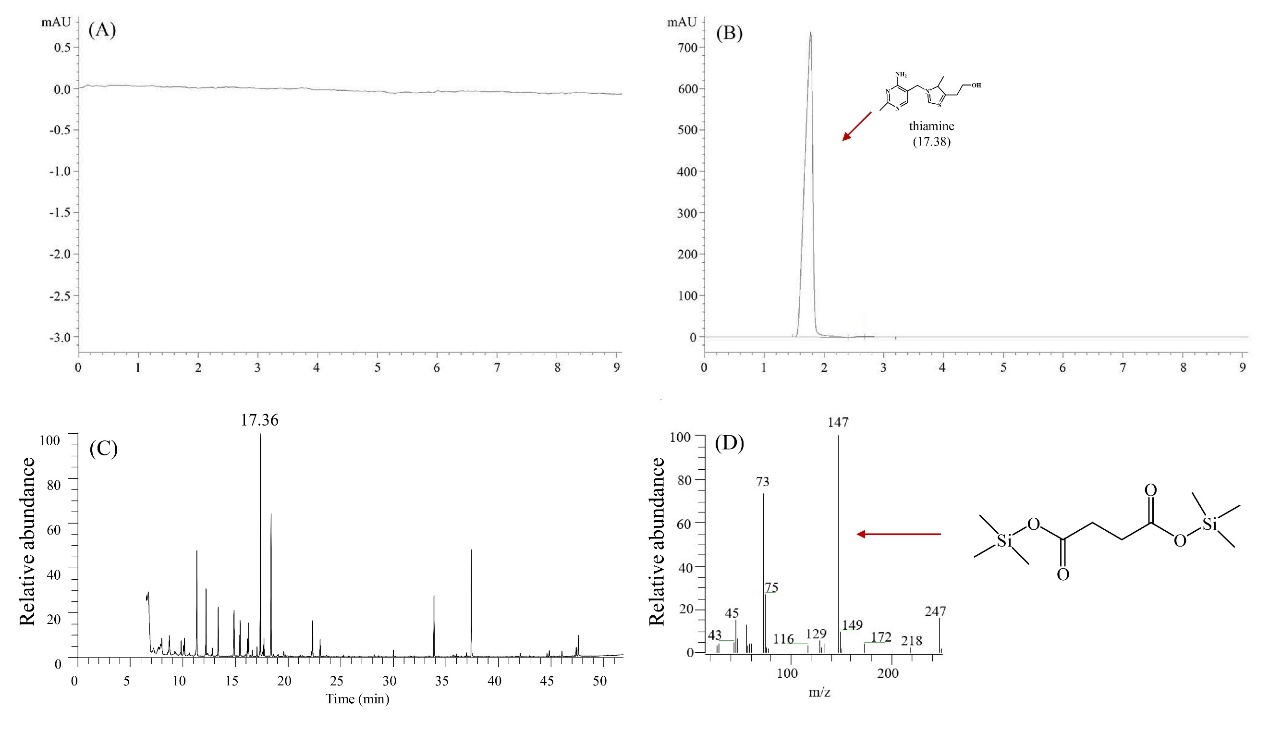


**Figure S10** HPLC profiles of the samples of the side without thiamine before (A) and after (B) one day shaking. (C) GC-MS profiles of the derivatized sample of the side without succinate after one day shaking. (D) GC-MS spectra of derivatized succinate in panel C.

**Figure S11** (A) Growth curve of strain K12 and K12Δ*thiE* in BSM with 20 mM succinate in the first transfer; (B) Growth curve of strain K12 and K12Δ*thiE* in BSM with 20 mM succinate and 0.01 mM thiamine in the first transfer; (C) Biomass of K12, K12Δ*thiE*, coculture of K12 and K12Δ*thiE* (the initial inoculum size of the total was OD_600_=0.06, and the cell ratios of two strains was 1:1) for 1 day without exogenous thiamine in different transfers. Error bars: SD from three independent replicates.


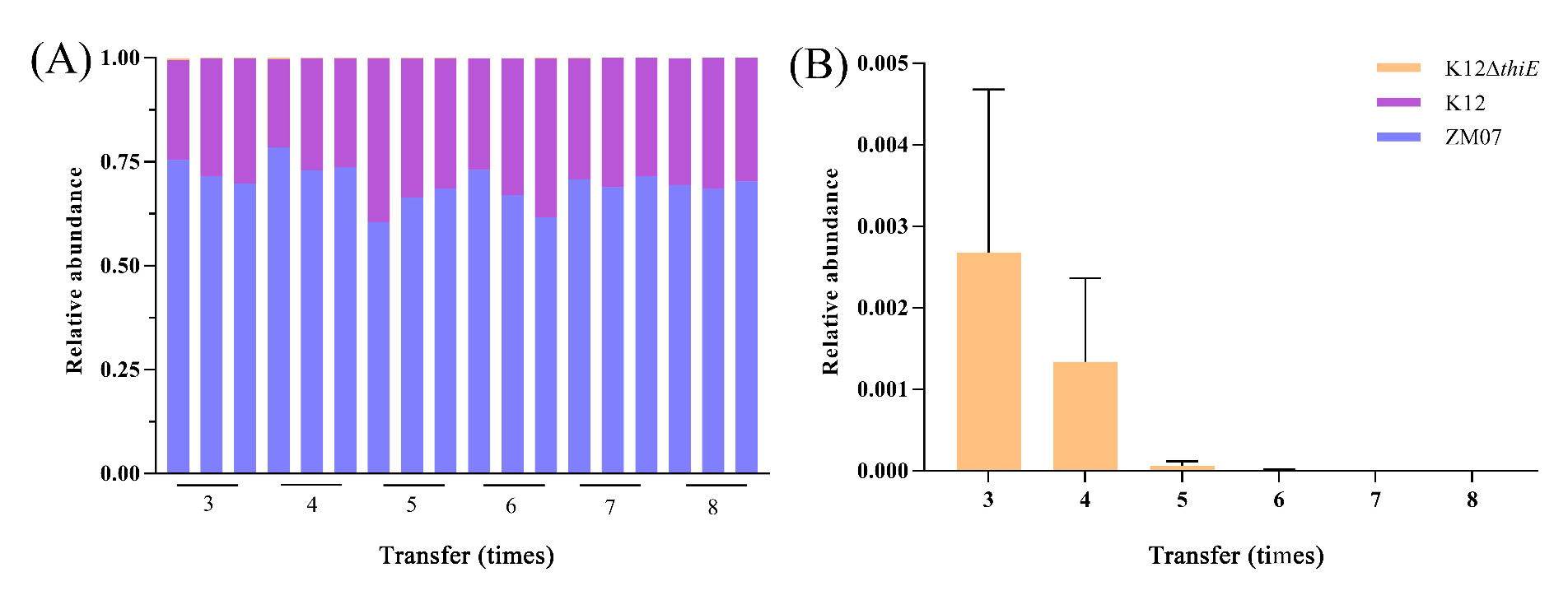


**Figure S12** (A) Relative abundances of ZM07, K12 and K12Δ*thiE* in a three-strain system (ZM07, K12 and a trace amount of K12Δ*thiE,* which was added in the 3^rd^ transfer to avoid the effects of intracellular thiamine on strain ZM07) in different transfers. (B) Detail of the relative abundance of K12Δ*thiE*. Error bars: SD from three independent replicates.


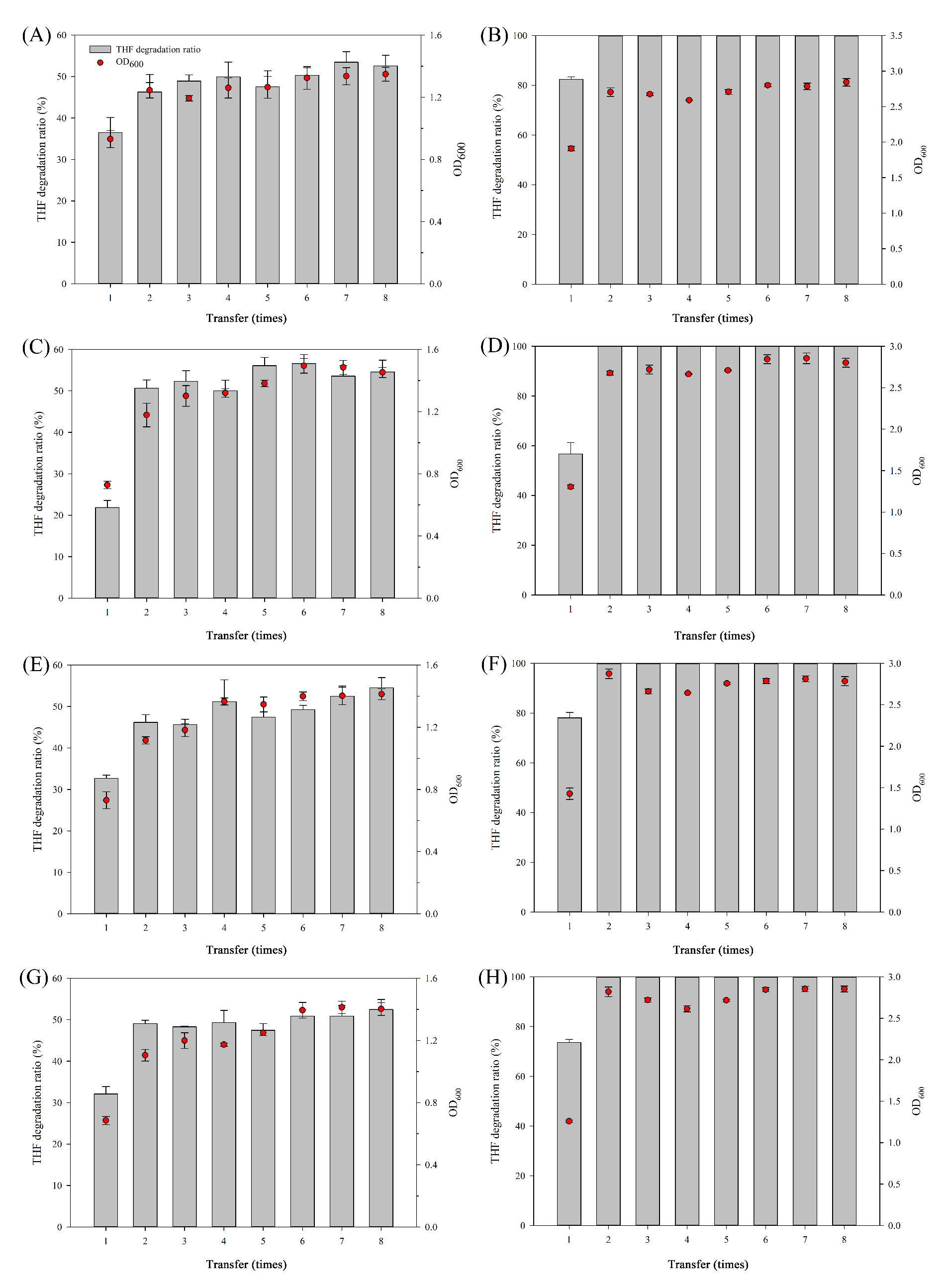


**Figure S13** THF degradation ratio and total biomass of the systems for 2 days (left) and 3 days (right) in thiamine-rich medium. (A) and (B) ZM07; (C) and (D) ZM07 and K12; (E) and (F) ZM07 and K12Δ*thiE*; (G) and (H) ZM07, K12 and K12Δ*thiE*. Error bars: SD from three independent replicates.
